# Interaction of porcine circovirus-like virus P1 capsid protein with host proteins

**DOI:** 10.1186/s12917-021-02926-6

**Published:** 2021-06-26

**Authors:** Libin Wen, Jiaping Zhu, Fengxi Zhang, Qi Xiao, Jianping Xie, Kongwang He

**Affiliations:** 1 Institute of Veterinary Medicine, Jiangsu Academy of Agricultural Sciences, Nanjing, Jiangsu China; 2 Key Laboratory of Animal Diseases, Diagnostics, and Immunology, Ministry of Agriculture, Nanjing, Jiangsu China; 3 Jiangsu Co-innovation Center for Prevention and Control of Important Animal Infections Diseases and Zoonoses, Yangzhou, Jiangsu China

**Keywords:** Porcine circovirus-like virus P1, yeast two-hybrid assay, Cap, cellular protein, co-immunoprecipitation

## Abstract

**Background:**

Porcine circovirus-like virus P1 is a relatively new kind of virus that is closely related to the post-weaning multisystemic wasting syndrome, congenital tremors, and abortions in swine. The molecular mechanisms of P1 virus infection and pathogenesis are fully unknown. To analyze P1 and its host interactions, we used a yeast two-hybrid (Y2H) assay to identify cellular proteins interacting with the Cap of the P1 virus. In this study, the Cap of the P1 virus exhibited no self-activation and toxicity to yeast cells and was used as bait to screen the Y2H library prepared from the pancreas tissue.

**Results:**

Five cellular proteins (EEP, Ral GDS, Bcl-2-L-12, CPS1, and one not identified) were found to interact with P1 Cap. The interaction between Cap and Ral GDS was confirmed by co-immunoprecipitation.

**Conclusions:**

Our data are likely to support the future investigation of the underlying mechanism of P1 infection and pathogenesis.

**Supplementary Information:**

The online version contains supplementary material available at 10.1186/s12917-021-02926-6.

## Background

Porcine circoviruses (PCVs), which belong to the *Circoviridae* family, are small icosahedral non-enveloped viruses with a small circular single-stranded DNA genome of approximately 2.0 kb. To date, four genotypes of PCV (PCV1, PCV2, PCV3, and PCV4) have been described [1–5]. Among them, PCV2, the most widely studied, is considered as the etiological agent of the post-weaning multisystemic wasting syndrome (PMWS). The clinical signs of PMWS are mainly characterized by progressive weight loss, diarrhea, anemia, and enlarged lymph nodes in post-weaned pigs approximately 8–12 weeks old. PMWS, first reported in Canada in 1991 and described in all regions of the world, has a severe impact on swine production worldwide [6]. However, PCV2 infection alone does not necessarily result in typical clinical symptoms of PMWS because the molecular basis of PCV2 pathogenicity is not fully understood [7].

Porcine circovirus-like virus P1 was first identified in China in 2005 in pigs with PMWS, and then in cattle, goats, rabbits, dogs, and cats [8–10]. The virion is icosahedral, non-enveloped, and 25 nm in diameter. Similar to PCV2, the genome of the P1 virus is also a circular single-stranded DNA molecule, but its size is only 648 nucleotides, about one-third the size of PCV2. In addition to the nucleotide sequence highly homologous to PCV2 ORF2, the viral genome also contains an exo-nucleotide fragment. The genome of the P1 virus consists of eight open reading frames, of which the largest ORF1 gene is located on the viral minus-strand and encodes the capsid protein of 12.5 kDa, the only structural protein of the virion and comprises the dominant immunogenic epitope of P1 [11, 12]. The ORF1 of the P1 strain originally reported encodes 114 amino acids. In 2017, an extended mutant of the ORF1 appeared that encodes 122 amino acids [13]. The N-terminal portion of the capsid protein (amino acids 1–83) is highly homologous with the corresponding amino acids of PCV2 ORF2, reaching 86 -100 %. However, the C-terminal 31 or 39 amino acids have low homology with PCV2 ORF2 because of a base deletion in the open reading frame leading to an encoding mutant. In 2020, three intermediate strains in the evolution of PCV2 and P1 appeared. The genome has a full length of 649 nucleotides, and its ORF1 encodes 163 amino acids. According to the evolutionary analysis at the nucleotide level, they have a close relationship with other P1 strains, while also having a close relationship with PCV2 based on the amino acid level of the capsid protein [14].

Experimentally inoculating conventional pigs with an infectious molecular clone of the P1 virus can cause PMWS-like symptoms. Affected pigs may exhibit skin pallor and diarrhea [15]. Also, the P1 virus is closely related to congenital tremor and abortion [16, 17]. Recently, the P1 virus was successfully used to infect Balb/c mice, and the virus was manifested by the appearance of specific antibodies, the replication of the virus, and microscopic lesions [18].

The interaction between the viral protein and host protein is an important clue to understanding the virus’s infection and pathogenesis. In this study, we have used a yeast two-hybrid approach for gaining a more detailed knowledge of the P1 virus-host interaction. Five porcine proteins that interacted with Cap of the P1 virus were screened. The physical interaction between P1 Cap and one of the porcine proteins was verified by co-immunoprecipitation *in vitro*.

## Methods

### Animal

A 5-day-old healthy suckling piglet was obtained from a PCV2-free breeding herd. The pigs were confirmed to be free of PCV2 and P1 infections with PCR for viral nucleic detection. The pig was euthanized by electric shock. After euthanasia, the pancreas was quickly removed and immediately frozen in liquid nitrogen for total RNA extraction.

### Extraction of total RNA, isolation of mRNA, and construction of the Y2H library

One gram of pancreatic tissue was ground into a powder with liquid nitrogen. Total RNA was extracted from the pancreatic tissue powders using the TRIzol method (Invitrogen, Carlsbad, CA, USA), and its quality was assessed by agarose gel electrophoresis. According to the manufacturer’s instructions, the mRNAs were purified using Oligotex mRNA kits (Qiagen, Hilden, Germany), as described in the manual, and cDNA was synthesized with Superscript II Reverse Transcriptase (Invitrogen). To ensure that the open reading frames of host proteins can be translated correctly, the 5’ ends of these cDNA fragments were added with connectors containing three open reading frames (ACAACTTTGTACAAAAAAGTTGG; ACAACTTTGTACAAAAAAGTTGGA; and ACAACTTTGTACAAAAAAGTTGGAA) separately.

Then, cDNA products were electrophoresed in 1 % low melting-point agarose gel, and cDNA fractions longer than 1,000 bp were recovered by gel recovery kit after cutting the gel slices, and precipitated with ethanol. The cDNAs were ligated into pGADT7 vectors (Clontech Laboratories) using infusion technology. The cDNA library was then electro-transformed into *Escherichia coli* DH10B competent cells (Invitrogen).

### Bait plasmid construction

For generating bait plasmids, ORF1 of the P1 virus was amplified from the P1 HB1 strain (GenBank accession no. EF514716) using PCR with specific primers containing *Sfi*I restriction enzyme sites (Forward primer: 5’-AAGGCC ATTAC GGCC ATGATGAGATTTAATATTGACG-3’, Reverse primer: 5-CCGGCC GAGGC GGCC TCAGCCAAAGCTGATTCCTTTTG-3’). The amplification of DNA was performed for 5 min at 98 °C, followed by 35 cycles of 30 s at 98 °C, 30 s at 55 °C, and 15 s at 72 °C, and a final extension of 5 min at 72 °C. The PCR products were purified using a DNA gel extraction kit (Axygen) and then digested with restriction enzyme *Sfi*I. The digested fragment was linked to the *Sfi*I-digested pGBKT7 vector (Clontech). The ligation products were transformed into *E.coli* TOP10 (Invitrogen) and sequenced. The resultant plasmids expressed the viral structure protein fused to the GAL4 DNA-binding domain and were used as bait proteins in yeast two-hybrid screens.

### Detection of self-activation of the P1 ORF1 bait protein

Self-activation of the bait protein was determined by cotransformation of pGADT7 and pGBKT7-ORF1. Simultaneously, the vectors pGADT7-largeT and pGBKT7-p53 were also cotransformed into yeast cell AH109 as a positive control, the vectors pGADT7-largeT and pGBKT7-lamin C as a negative control, and then the yeast cells were plated on SD medium lacking tryptophan and leucine (SD/-Trp/-Leu, SD-TL) agar plates to screen the positive clones. Six colonies were randomly selected from the yeast transformants from the total cotransformation of pGADT7 and pGBKT7-ORF1 for the self-activation test. The detection of three reporter genes, namely, *HIS3, ADE2* and *lacZ*, was included. The *HIS3* and *ADE2* reporter genes were identified on SD/-Trp/-Leu/-His/-Ade (SD-TLHA) agar plates, and the *lacZ* gene was identified by β-galactosidase assays.

### Yeast two-hybrid screening procedure

The Matchmaker Gal4 Two-Hybrid System 3 (Clontech) was used according to the Yeast Protocols Handbook (PT3024-1) to detect interactions between Cap encoded by the P1 virus and porcine cellular proteins. The complete coding sequences of the Cap of the P1 virus were cloned into pGBKT7 and used as bait to screen a cDNA library prepared from the pancreas of a healthy, non-PMWS-affected pig in pGADT7.

Yeast strain AH109 transformants containing the pGBKT7-ORF1 bait plasmid were used to prepare competent cells as the recipients. The prey plasmids (pGADT7 cDNA library) were transformed and plated on SD/-Trp/-Leu/-His/+5mM 3-AT medium at 30 °C for 3 days. After 3 days of culture, the colonies were screened by a replica-plating method with flannelette, and then the culture was continued for 7–14 days to eliminate the interference of the background growth colonies. Colonies with a diameter > 2 mm were selected as primary positive clones and plated on SD/-Trp/-Leu for 2–3 days to analyze reporter genes (*HIS3, ADE2*, and *LacZ*).

The screened positive clones were implanted in SD/-Trp/-Leu liquid medium, and the yeast plasmids were extracted using the Yeast Plasmid Extraction Kit (Solarbio, China) according to the manufacturer’s instructions after overnight shaking culture. *E. coli* Top10 cells were transformed with prey plasmids and grown on LB agar plates. The positive transformants were transferred to LB liquid culture containing ampicillin, and then prey plasmids were extracted using the AxyPrep Plasmid Miniprep Kit (Axygen, China). The plasmids were sequenced and analyzed using the NCBI BLASTP program (https://blast.ncbi.nlm.nih.gov).

The respective positive prey plasmids were transformed into yeast transformant AH109 containing pGBKT7-ORF1 bait plasmid and selected on SD/-Trp/-Leu plates for report genes (*HIS3, ADE2*, and *LacZ*) analysis to confirm the results.

### Co-immunoprecipitation (Co-IP)

Co-immunoprecipitation (Co-IP) assay was mainly carried out as previously described [19]. HEK293T cells were cultured with 5 % CO_2_ and at 37 °C in Dulbecco’s modified Eagle’s medium (DMEM) supplemented with 10 % (v/v) fetal bovine serum, 100 U/mL penicillin, and 100 µg/mL streptomycin. Transfection was performed using Lipofectamine 2000 reagent (Invitrogen) according to the manufacturer’s instructions. The cells in 6-well plates were simultaneously transfected with pcDNA3.1-Ral guanine nucleotide dissociation stimulator-Flag based constructs expressing Flag-tagged cellular proteins and the respective pcDNA3.1-ORF1-His constructs expressing His-tagged viral Cap or His as control. Simultaneously, a comparative experiment was set up. The cells in 6-well plates were also simultaneously transfected with pcDNA3.1-ORF1-His expressing His-tagged viral Cap and the pcDNA3.1-Ral guanine nucleotide dissociation stimulator-Flag based constructs expressing Flag-tagged cellular proteins or Flag as control. Cells were washed with PBS and incubated on ice for 30 min in a lysis buffer (20 mM Tris/HCl, pH7.5, 150 mM NaCl, 0.1 % SDS, 1 mM EDTA, 1 % NP40) containing one tablet of complete mini protease inhibitor (Roche) and 100 µL phosphatase inhibitor cocktail (Roche) per 10 mL at 48 h post-transfection. Soluble proteins were subjected to immunoprecipitation with anti-His Tag Rabbit Polyclonal Antibody or Anti-Flag Tag Mouse Monoclonal Antibody (Abbkine) at 4 °C overnight. After the addition of 10 µL protein A Sepharose (Merck Millipore) for2–4 h at 4 °C, the adsorbates were washed four times with an ice-cold lysis buffer and subjected to sodium dodecyl sulfate polyacrylamide gel electrophoresis (SDS-PAGE). Aliquots of total cell lysate (50 µL) were included as input controls. After SDS-PAGE, proteins were blotted onto PVDF membranes (Bio-Rad Laboratories, USA). The membranes were blocked with 5 % milk powder in PBS and 0.05 % Tween 20 (PBS-T) for 1 h at room temperature and probed with a primary antibody in PBS-T containing 5 % milk powder overnight at 4 °C. Membranes were washed three times with PBS-T and treated with secondary antibody diluted in PBS-T containing 5 % milk powder (horseradish peroxidase (HRP)-conjugated goat anti-mouse antiserum (Jackson) diluted at 1:4,000, and HRP-conjugated goat anti-rabbit antiserum diluted at 1:2,000) for 1 h at room temperature. The membranes were washed three times for 10 min each, and antigen–antibody complexes were visualized by chemiluminescence using the ECL–Western blotting detection system (GE).

## Results

### Characteristics of the cDNA library

**Fig. 1:**
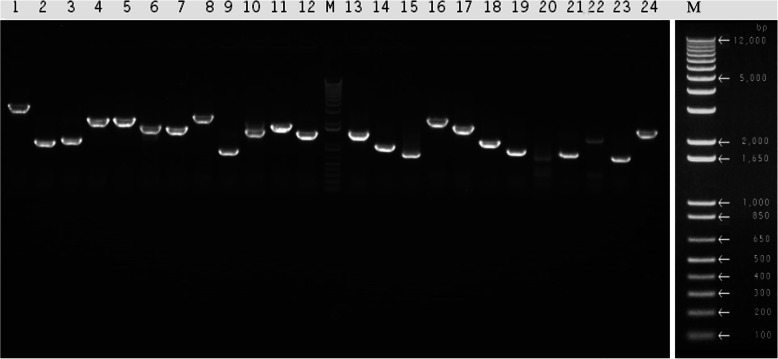
PCR products of the 24 randomly selected clones in 1.0 % agarose gel electrophoresis. DL12000 (Takara) was used as the marker. An enlarged marker is shown on the right side of the figure. The number of the lanes indicates the 24 randomly selected clones

A high-quality cDNA library of piglet pancreatic tissues was constructed in this study. The titer of the library was counted for 1 × 10^7^ CFU (the total size of the library = the number of colonies on the plate/10 µL×100 × 1 × 10^3^ µL×total volume of the microbial library). The inserts were found in 98 % of the tested colonies, and the average length of the inserts, based on the randomly selected 24 recombinant plasmids in the library, was approximately 1,500 bp (ranging from 500 bp to 3,000 bp) as measured by PCR amplification (Fig. 1), which indicated that the cDNA library met the requirements for follow-up analysis.

### Testing bait for toxicity and auto activation

**Fig. 2:**
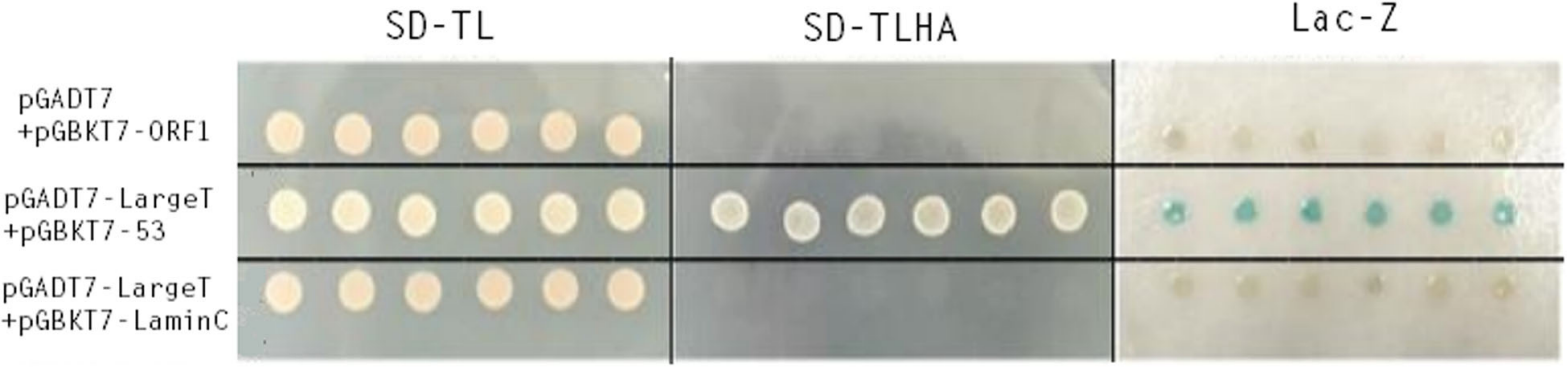
Detection of self-activation of the bait protein. The AH109 yeast cells that were cotransformed with pGADT7 and pGBKT7-ORF1, pGADT7-LargeT and pGBKT7-p53 (as a positive control), pGADT7-LargeT and pGBKT7-laminC (as a negative control), were plated on SD-TL and SD-TLHA medium for the auto activation test. Yeast co-transfected with plasmids pGADT7 and pGBKT7-ORF1 cannot grow on SD-TLHA medium and did not turn blue in the β-galactosidase assay, indicating that pGBKT7-ORF1 does not autonomously activate the reporter genes in yeast cells without a prey protein. The images were cropped, and full-length images are included in Supplementary Fig. 2a to 2c

The bait gene was subcloned into the GAL4 DNA-binding fusion vector pGBKT7. The DNA-binding construct (pGBKT7-ORF1) and various plasmids were cotransformed into yeast strain AH109. The yeast strains could grow normally on the SD-TL plate, while only the positive control could grow on the SD-TLHA plate. As the negative control, six colonies randomly selected from pGBKT7-ORF1 and pGADT7 co-transformants could not grow on the SD-TLHA plate, and the *lacZ* test results were the same as those of the negative control. Thus, autoactivation of the P1 ORF1 bait clone was excluded (Fig. 2).

### Screening of P1 Cap-interacting proteins

**Fig. 3:**
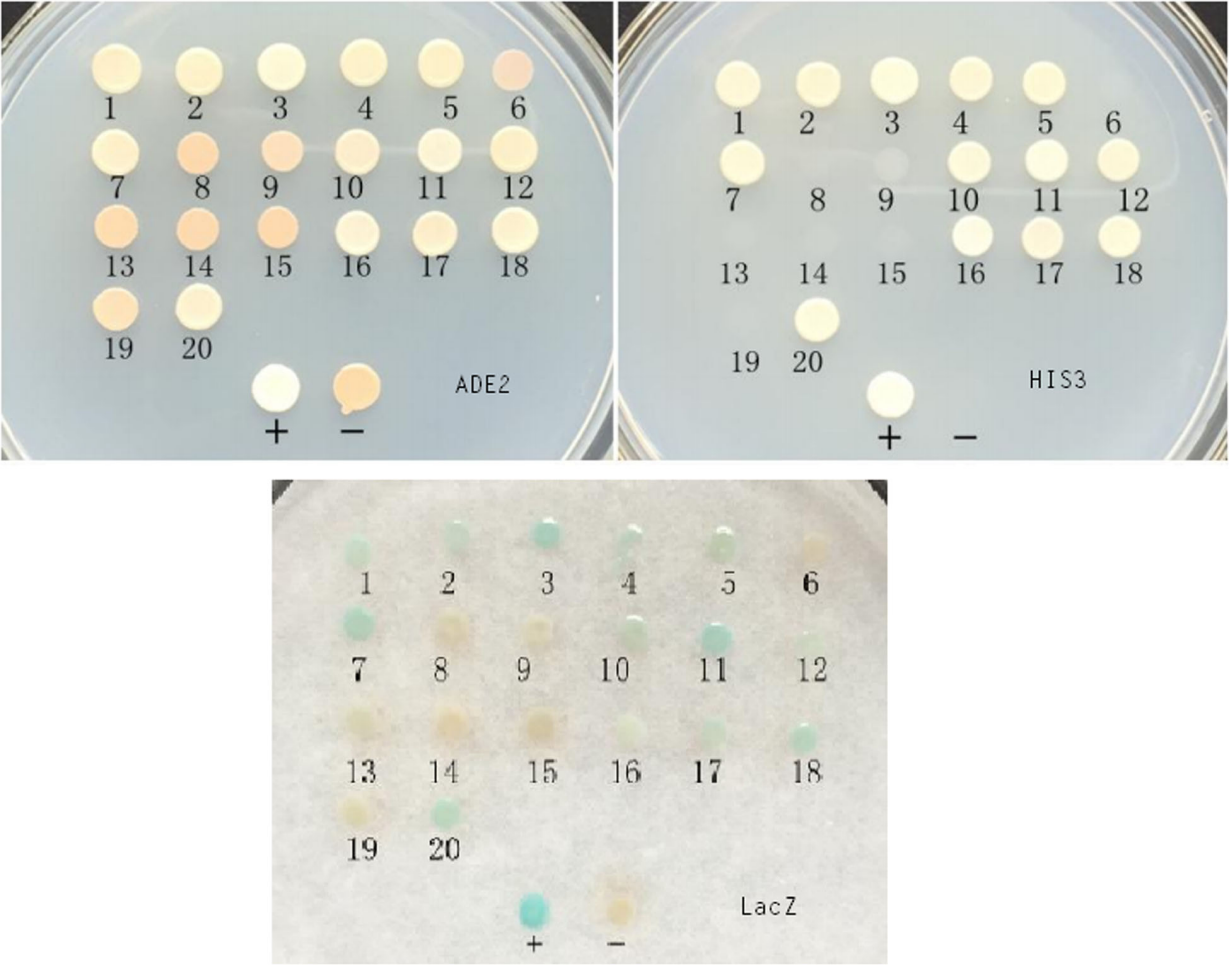
Yeast two-hybrid assay with P1 Cap gene products. The 20 initial positive clones (1 to 20) were inoculated into SD-TL and SD-TLHA medium and analyzed for lacZ reporter gene expression. The 13 clones (1–5, 7, 10–12, 16–18, and 20) can grow on SD-TLHA medium and turn blue in the β-galactosidase assay, indicating that these clones are real positive clones

**Fig. 4:**
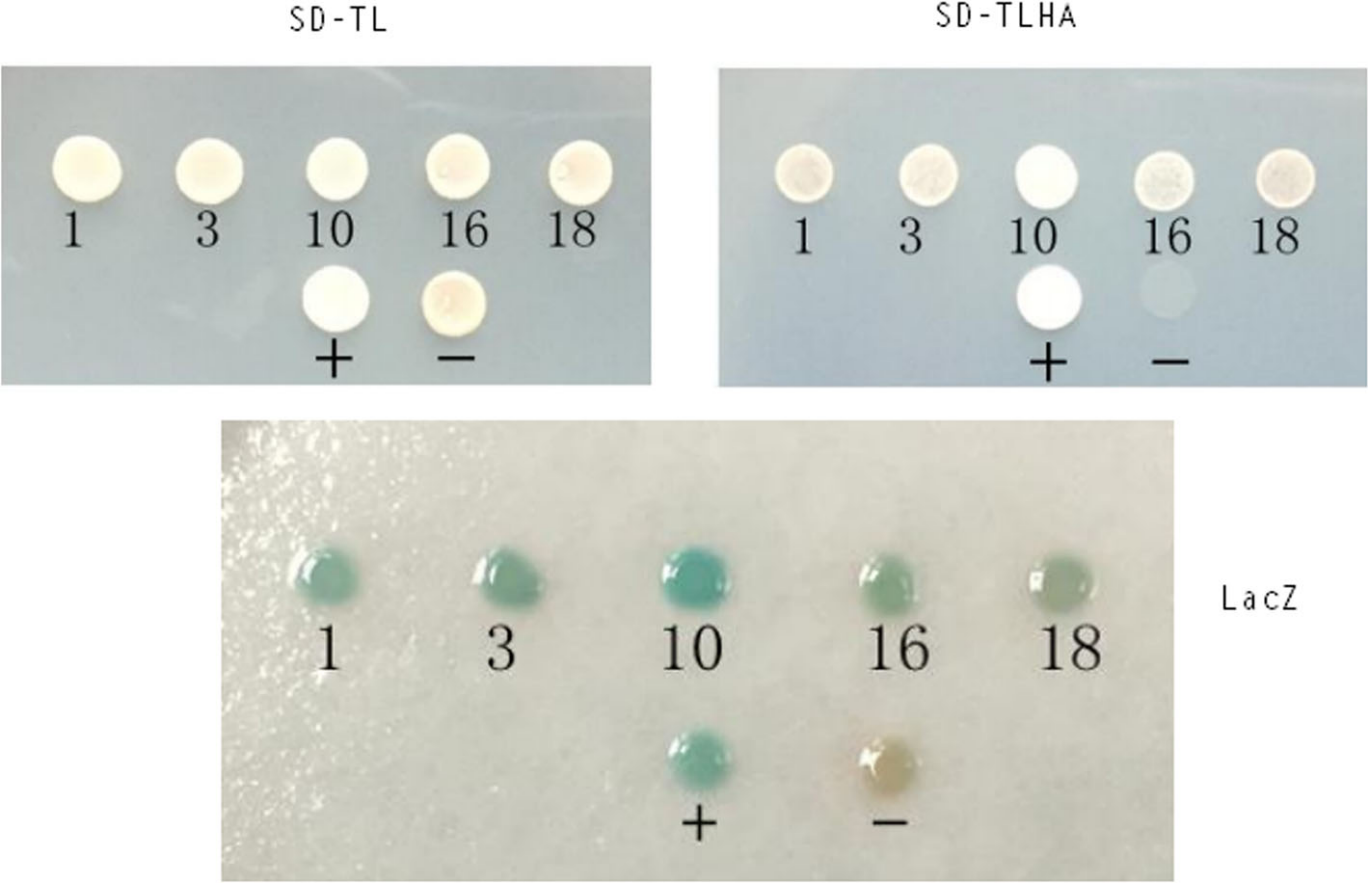
Retransformation validation of representative five host genes interacting with P1 Cap gene products in yeast two-hybrid system. The five clones (1, 3, 10, 16, and 18) were inoculated into SD-TL and SD-TLHA medium plates and analyzed for *lacZ* expression. The five clones that grew on SD-TLHA medium and turned blue in the yeast were positive. The images were cropped, and full-length images are included in Supplementary Fig. 4a to 4c

A cDNA library from the pancreas of a healthy pig was used in a yeast two-hybrid assay to screen cellular proteins that interact with the Cap of the P1 virus. P1 Cap was used as a bait protein to screen approximately 1 × 10^7^ cDNA library clones. A total number of 20 primary positive clones grew. The 20 initial positive clones grown on the SD-TL plates were diluted with sterile water and inoculated into SD-TL and SD-TLHA plates, and cultured at 30 °C for 3–4 days to exclude false-positive clones. The AH109 strain could not synthesize *His* and adenine (*Ade*), and it is therefore unable to grow on a medium lacking either of the two essential amino acids. Only when bait and prey proteins interact, Gal4-responsive *His3* and *Ade2* expression allows the cell to biosynthesize *His3* and *Ade2* and grow on –His–Ade minimal medium. Finally, 13 true interacting clones (clones 1–5, 7, 10–12, 16–18, and 20) representing 5 genes (clones 1, 2, 4, 5, 12, 17; clones 3, 11; clones 7, 18, 20; clone 10; and clone 16) were identified through screening, sequenced and analyzed by a BLAST search for homology to porcine proteins (Fig. 3). Prey plasmids encoding five putative interacting partners were retransformed into yeast strain AH109 with the bait plasmid pGBKT7-ORF1 to verify the interaction. Transformed colonies growth on SD-TL and SD-TLHA plates and growth of blue colonies expressing *LacZ* demonstrated physical interactions between the encoded proteins, and five positive clones (clones 1, 3, 10, 16, and 18) were identified by retransformation in yeast (Fig. 4). Five cellular proteins interacted with the P1 Cap: the endonuclease/exonuclease/phosphatase family protein (EEP); a guanine nucleotide dissociation stimulator of Ral protein (Ral GDS); the bcl-2-like protein 12; the carbamoyl-phosphate synthase 1(CPS1). Another clone did not show any indicative similarity with any known protein by BLAST searches (Table 1).

**Table 1:** Identified cDNA clones coding for P1 Cap-interacting proteins

Number of cDNA clones	Sequence ID	Best hits of homolog proteins identified in databases
6	LT634572.1 *E value*= 7e-52	endonuclease/exonuclease/phosphatase family protein E value =1e-14
2	XM_008761632.2 *E value* =6e-151	A Guanine Nucleotide Dissociation Stimulator Of Ral Protein E value =4e-66
3	CP027092.1 *E value* =7e-28	Not hit
1	CU633284.7 *E value*= 1e-19	Bcl-2 like protein 12E value =3e-04
1	XM_005672159.3 *E value *=2e-80	Sus scrofa carbamoyl-phosphate synthase 1

### Confirming the interaction by Co-IP

**Fig. 5:**
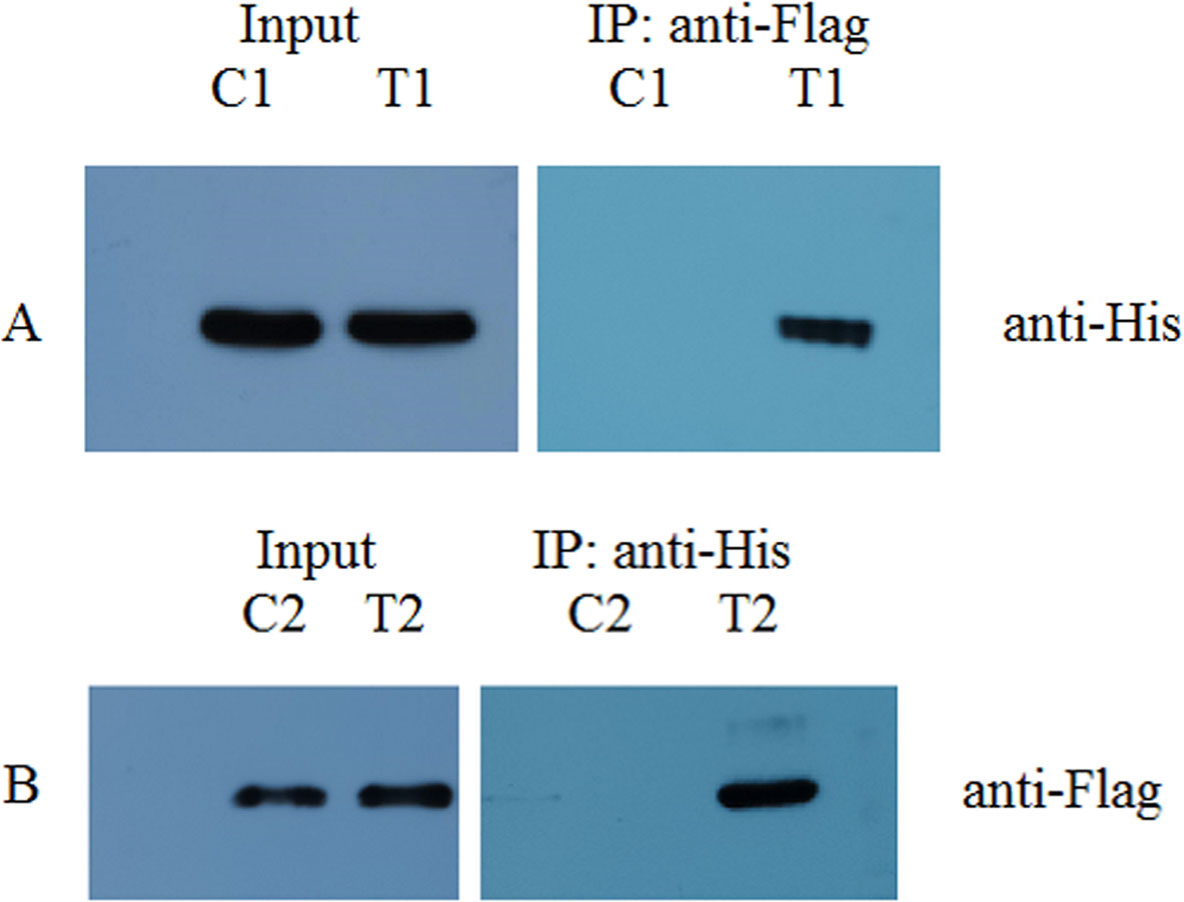
Co-immunoprecipitation of Cap interacted with Ral GDS. HEK 293 Graham cells were co-transfected with pcDNA3.1-Ral GDS-FLAG and pcDNA3.1-His (C1); pcDNA3.1-Ral GDS-FLAG and pcDNA3.1-ORF1-His (T1); pcDNA3.1-FLAG and pcDNA3.1-ORF1-His (C2); and pcDNA3.1-Ral GDS-FLAG and pcDNA3.1-ORF1-His (T2). The immune complexes were incubated with anti-FLAG (**A**) or anti-His antibodies (**B**), and then pre-coupled to protein A agarose beads and subjected to Western blotting analysis. The gels/blots were cropped, and full-length blots/gels are presented in Supplementary Fig. 5a to 5d. The cropped Fig. 5 A comes from the Supplementary Fig. 5a and 5b; and B comes from the Supplementary Fig. 5c and 5d

Co-IP was used to verify the physical interaction between P1 Cap and the cellular target protein (Ral GDS). Here, the interaction between P1 ORF1 and a selected host protein Ral GDS was studied in 293T cells. The ORF1 and Ral GDS genes were inserted into the eukaryotic expression vector pCDNA3.1. A specific co-immunoprecipitation of His-ORF1 and Flag-Ral GDS was observed with either anti-His antibody or anti-Flag antibody (Fig. 5). The results showed that the cellular protein Ral GDS functionally interacts with P1 Cap.

## Discussion

Clinically, the P1 virus can infect animals alone or with PCV2, which indicates that P1 virus is not a replication defective virus of PCV2. At present, the dependency of the P1 viral life cycle in host cells, including viral attachment, entry, assembly, and release, is still poorly understood. Little is known about the cellular events caused by infection with P1 virus. The study showed that several porcine genes, such as MMP2, were down-regulated not only in tissues of infected pigs and mice but also in ST cells and that the virus induces the Wnt signaling pathways [20].

The Y2H system, a practical tool originally developed by Field and Song to identify new protein interacting partners for a protein of interest, has been widely used in various organisms, including animals, plants, and fungi [21].

The molecular mechanism of P1 viral infection and pathogenesis, depending on the interaction between the virus and host proteins is still poorly understood. The pancreas is an important endocrine organ that secretes several hormones, such as insulin, glucagon, and digestive enzymes that can digest protein, fat, and sugar, closely related to animal growth and development. In an earlier study, we have demonstrated that the P1 capsid protein participates in the pancreatic secretion signaling pathway [22]. Therefore, in this study, we constructed a porcine pancreas cDNA library for screening host proteins interacting with Cap of P1 virus by Y2H assay.

Four definite porcine proteins were identified to bind to the P1 Cap. EEP has endonuclease activity, exonuclease activity, and hydrolysis activity of nucleic acid phosphodiester bond. It has been reported to regulate cellular cholesterol efflux by controlling cellular levels and activity of ATP-binding cassette transporter A1 (ABCA1) via endonuclease–exonuclease–phosphatase family domain containing 1 (EEPD1) [23].

Ral GDS, a guanine nucleotide dissociation stimulator for Ral, has GTPase regulator activity, guanyl-nucleotide exchange factor activity, and catalytic activity regulation. It is a member of the Ras GTPase superfamily that regulates cellular proliferation, differentiation, and transformation by mediating multiple signal transduction pathways. For example, it can mediate skeletal myogenesis and cytoskeletal reorganization [24]. The study has shown that the capsid protein of the porcine circovirus-like virus P1 participates in the signal pathway of pancreatic secretion, which affects the expression of pancreatic amylases, pancreatic proteases, and pancreatic lipases, thereby affecting the digestive functions of the body. That RAL GDS participates in the RAS- and RAP1 signaling pathways has been confirmed. A downstream effector of RAL GDS, the RAC, is also involved in the pancreatic secretion signaling pathway. Whether Cap of porcine circovirus-like virus P1 interacts with RAL GDS to regulate RAC expression and then participates in pancreatic secretion remains to be further studied.

Bcl-2-like protein 12, like other Bcl-2 family proteins, is a regulator of the mitochondrial apoptotic pathway in normal physiological and pathological states. Bcl-2 L12 is involved in the inhibition of cysteine-type endopeptidase activity associated with the apoptotic process, the negative regulation of intrinsic apoptotic signaling pathway in response to DNA damage by the p53 class mediator, and regulation of extrinsic apoptotic signaling pathway [25, 26]. Studies have shown that porcine circovirus-like virs P1 infection can induce the apoptosis of a large number of cells in immune organs (such as spleen, lymph node, tonsil) of pigs, resulting in the decline of immune function. The molecular mechanism of apoptosis caused by the P1 virus is unclear, Whether Cap of porcine circovirus- like virus P1 interacts with Bcl-2 to participate in apoptosis needs to be further studied.

CPS1 has catalytic activity, and its function is involved in the urea cycle, where it plays an important role in removing excess ammonia from the cell and involved in multiple signal transduction pathways, such as the biosynthesis of amino acids, metabolism of amino acids and derivatives, metabolism of polyamines, carbon metabolism, and nitrogen metabolism [27, 28]. The interaction between Cap of porcine circovirus-like virus P1 and CPS1 may affect the above-mentioned synthetic and metabolic processes and then affect the growth and development of the body.

With a yeast two-hybrid approach, Finsterbusch et al. have identified six cellular proteins (MKRN1, gC1qR, Par-4, NAP1, NPM1, and Hsp40) interacting with the Cap of PCV2 [29]. The bacterial two-hybrid assay revealed that the C1qB and P-selectin interact with PCV2 Cap [30]. Recently, up to 222 putative PCV2 Cap-interacting host proteins potentially involved in protein binding, DNA transcription, metabolism, and innate immune responses were identified in infected porcine kidney (PK-15) cells by coimmunoprecipitation combined with liquid chromatography mass spectrometry (LC-MS) approach [31]. The four host proteins interacting with the P1 Cap in this study were not included in the above-mentioned host proteins interacting with PCV2 Cap, although the amino acid sequences of the two viruses’ capsid proteins are highly homologous.

## Conclusions

In this study, a high-quality cDNA library from pancreatic tissue of a healthy piglet was constructed for screening proteins that interact with the P1 Cap in this study. Using the Y2H system, we have identified four definite porcine proteins interacting with the P1 Cap. Most of the annotated proteins perform multiple functions. Thus, the functions of the described interacting proteins regarding P1 infection and pathogenicity need further study. In any case, this study’s results will help us understand the infection and pathogenesis of the P1 virus.

## Supplementary Information

**Additional file 1: Figure S2.** Detection of self-activation of the bait protein. TheAH109 yeast cells that were cotransformed with pGADT7 and pGBKT7-ORF1, pGADT7-LargeT and pGBKT7-p53 (as a positive control), pGADT7-LargeT and pGBKT7-laminC (as a negative control), were plated on SD-TL (2a) and SD-TLHA medium (2b) fortheauto activation test.Yeast co-transfected with plasmids pGADT7 and pGBKT7-ORF1 cannot grow on SD-TLHA medium and did not turn blue intheβ-galactosidase assay, indicating that pGBKT7-ORF1 does not autonomously activatethereporter genes in yeast cells without a preyprotein (2c).

**Additional file 2: Figure S4.** Retransformation validation of representative five host genes interacting with P1 Cap gene products in yeast two-hybrid system. Thefive clones (1, 3, 10, 16, and 18 (repeat)) were inoculated into SD-TL (4a)and SD-TLHA medium plates (4b) and analyzed for *lacZ* expression. Thefive clones that grew on SD-TLHA medium and turned blue intheyeast were positive (4c).

**Additional file 3: Figure S5** Co-immunoprecipitation of Cap interacted with RalGDS. HEK 293 Graham cells were co-transfected with pcDNA3.1-RalGDS-FLAG and pcDNA3.1-His (5a); pcDNA3.1-RalGDS-FLAG and pcDNA3.1-ORF1-His (5b); pcDNA3.1-FLAG and pcDNA3.1-ORF1-His (5c); and pcDNA3.1-RalGDS-FLAG and pcDNA3.1-ORF1-His (5d).

## Data Availability

The datasets used and/or analysed during the current study available from the corresponding author on reasonable request.

## Abbreviations

PCV: Porcine circovirus
P1: Porcine circovirus like virus P1
Y2H: Yeast two-hybrid
Co-IP: Co-immunoprecipitation
EEP: The endonuclease/exonuclease/phosphatase family protein
Ral GDS: A guanine nucleotide dissociation stimulator of Ral protein
CPS1: The carbamoyl-phosphate synthase 1
