# Supplementary figures and images for: Interaction of porcine circovirus-like virus P1 capsid protein with host proteins
Source: BMC Vet Res. 2021 Jun 26;17:227. doi: 10.1186/s12917-021-02926-6 (PMC8235626; doi:10.1186/s12917-021-02926-6)

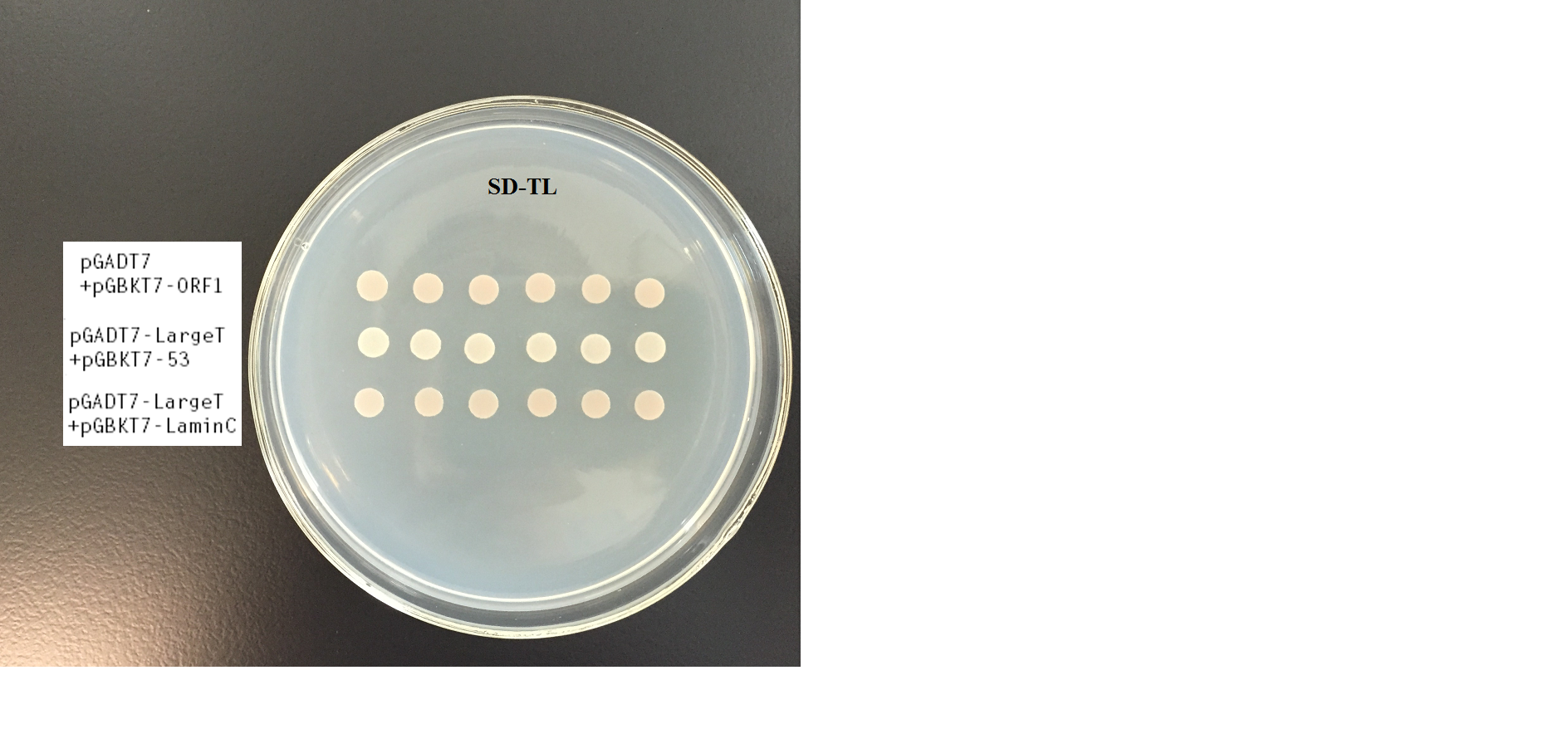

Supplement: Supplementary file 1 — Additional file 1: Figure S2. Detection of self-activation of the bait protein. TheAH109 yeast cells that were cotransformed with pGADT7 and pGBKT7-ORF1, pGADT7-LargeT and pGBKT7-p53 (as a positive control), pGADT7-LargeT and pGBKT7-laminC (as a negative control), were plated on SD-TL (2a) and SD-TLHA medium (2b) fortheauto activation test.Yeast co-transfected with plasmids pGADT7 and pGBKT7-ORF1 cannot grow on SD-TLHA medium and did not turn blue intheβ-galactosidase assay, indicating that pGBKT7-ORF1 does not autonomously activatethereporter genes in yeast cells without a preyprotein (2c). [file 12917_2021_2926_MOESM1_ESM.zip › Figure 2a.tif]

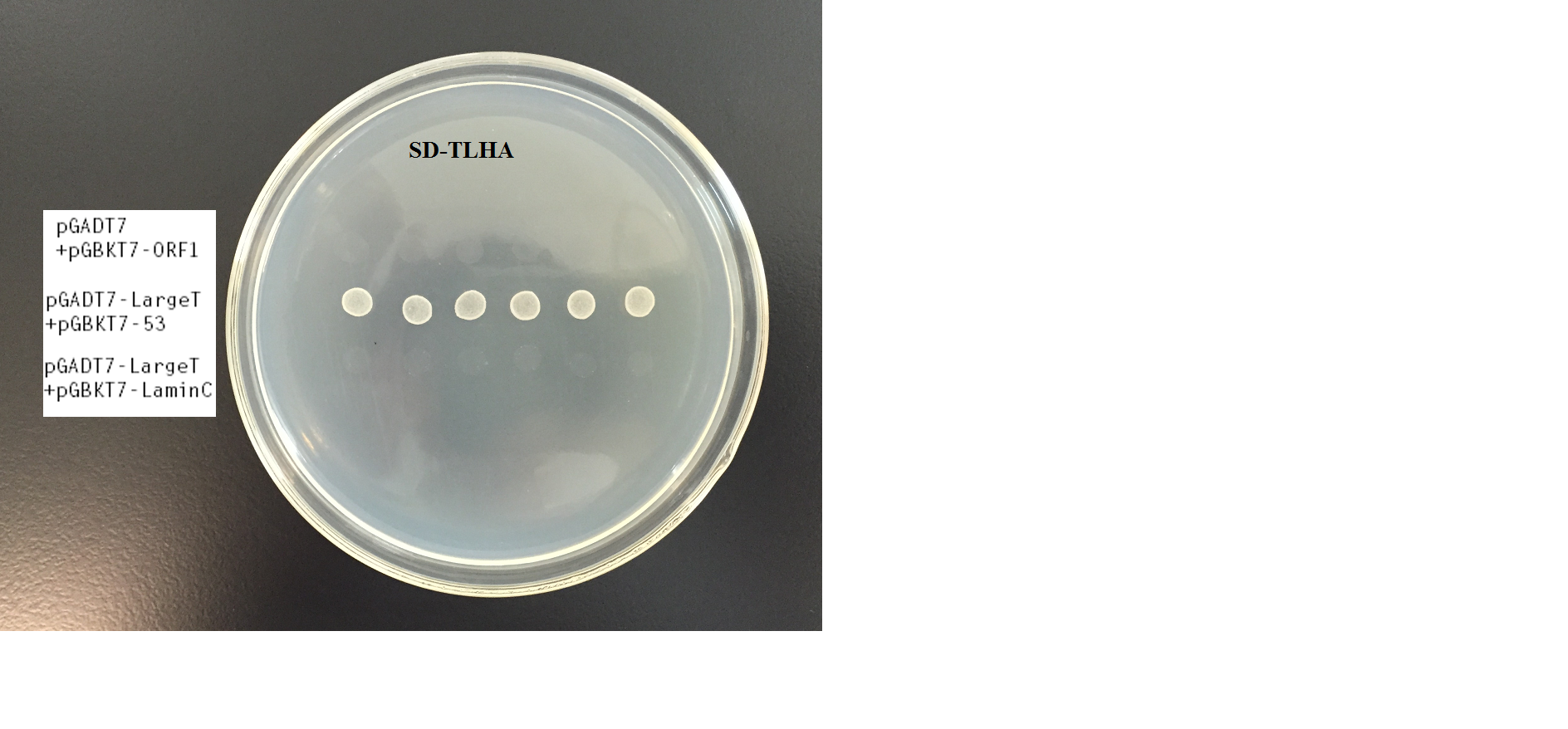

Supplement: Supplementary file 1 — Additional file 1: Figure S2. Detection of self-activation of the bait protein. TheAH109 yeast cells that were cotransformed with pGADT7 and pGBKT7-ORF1, pGADT7-LargeT and pGBKT7-p53 (as a positive control), pGADT7-LargeT and pGBKT7-laminC (as a negative control), were plated on SD-TL (2a) and SD-TLHA medium (2b) fortheauto activation test.Yeast co-transfected with plasmids pGADT7 and pGBKT7-ORF1 cannot grow on SD-TLHA medium and did not turn blue intheβ-galactosidase assay, indicating that pGBKT7-ORF1 does not autonomously activatethereporter genes in yeast cells without a preyprotein (2c). [file 12917_2021_2926_MOESM1_ESM.zip › Figure 2b.tif]

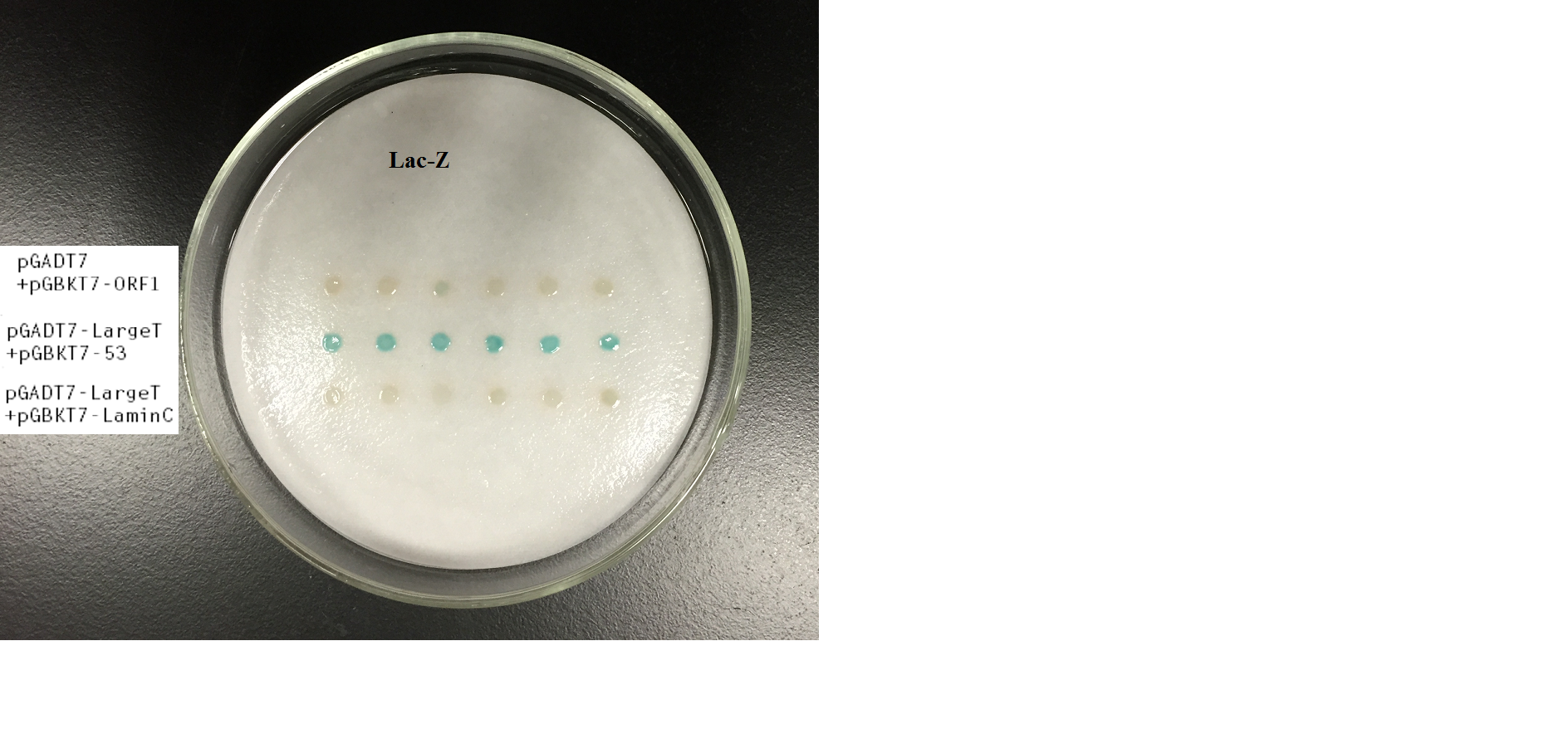

Supplement: Supplementary file 1 — Additional file 1: Figure S2. Detection of self-activation of the bait protein. TheAH109 yeast cells that were cotransformed with pGADT7 and pGBKT7-ORF1, pGADT7-LargeT and pGBKT7-p53 (as a positive control), pGADT7-LargeT and pGBKT7-laminC (as a negative control), were plated on SD-TL (2a) and SD-TLHA medium (2b) fortheauto activation test.Yeast co-transfected with plasmids pGADT7 and pGBKT7-ORF1 cannot grow on SD-TLHA medium and did not turn blue intheβ-galactosidase assay, indicating that pGBKT7-ORF1 does not autonomously activatethereporter genes in yeast cells without a preyprotein (2c). [file 12917_2021_2926_MOESM1_ESM.zip › Figure 2c.tif]

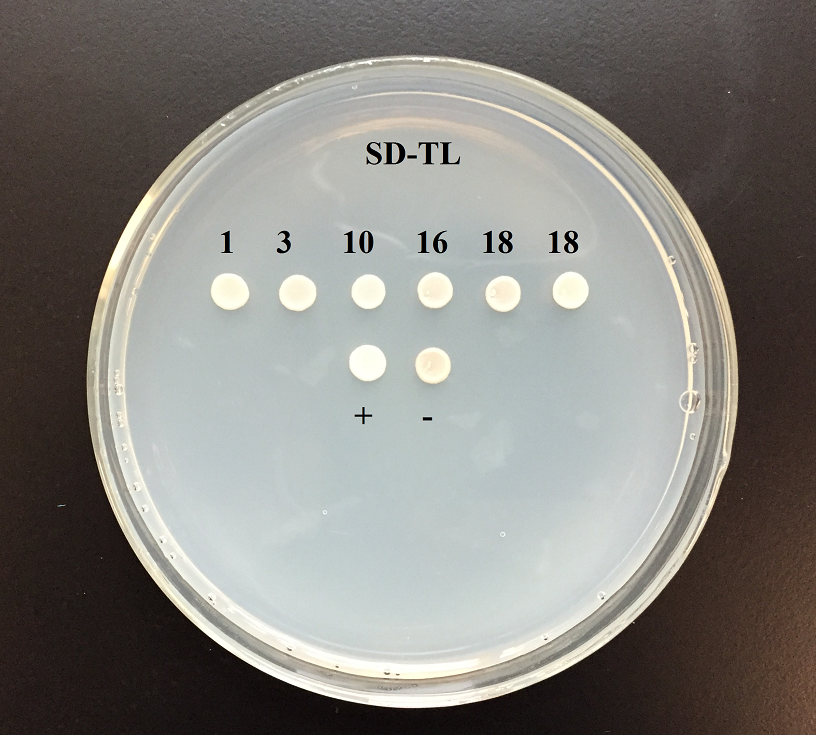

Supplement: Supplementary file 2 — Additional file 2: Figure S4. Retransformation validation of representative five host genes interacting with P1 Cap gene products in yeast two-hybrid system. Thefive clones (1, 3, 10, 16, and 18 (repeat)) were inoculated into SD-TL (4a)and SD-TLHA medium plates (4b) and analyzed for lacZ expression. Thefive clones that grew on SD-TLHA medium and turned blue intheyeast were positive (4c). [file 12917_2021_2926_MOESM2_ESM.zip › Figure 4a.tif]

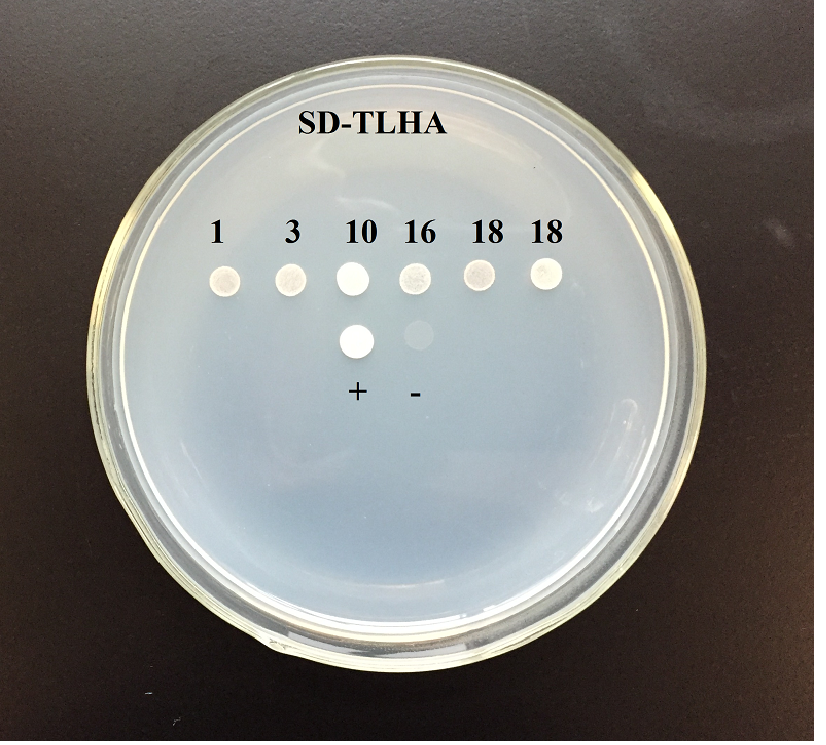

Supplement: Supplementary file 2 — Additional file 2: Figure S4. Retransformation validation of representative five host genes interacting with P1 Cap gene products in yeast two-hybrid system. Thefive clones (1, 3, 10, 16, and 18 (repeat)) were inoculated into SD-TL (4a)and SD-TLHA medium plates (4b) and analyzed for lacZ expression. Thefive clones that grew on SD-TLHA medium and turned blue intheyeast were positive (4c). [file 12917_2021_2926_MOESM2_ESM.zip › Figure 4b.tif]

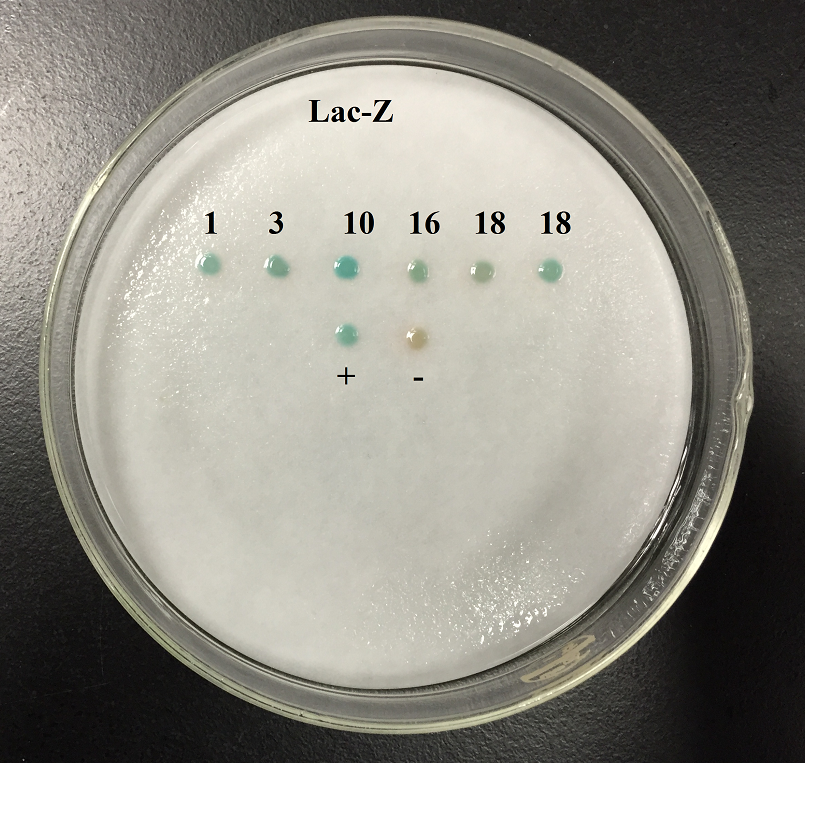

Supplement: Supplementary file 2 — Additional file 2: Figure S4. Retransformation validation of representative five host genes interacting with P1 Cap gene products in yeast two-hybrid system. Thefive clones (1, 3, 10, 16, and 18 (repeat)) were inoculated into SD-TL (4a)and SD-TLHA medium plates (4b) and analyzed for lacZ expression. Thefive clones that grew on SD-TLHA medium and turned blue intheyeast were positive (4c). [file 12917_2021_2926_MOESM2_ESM.zip › Figure 4c.tif]

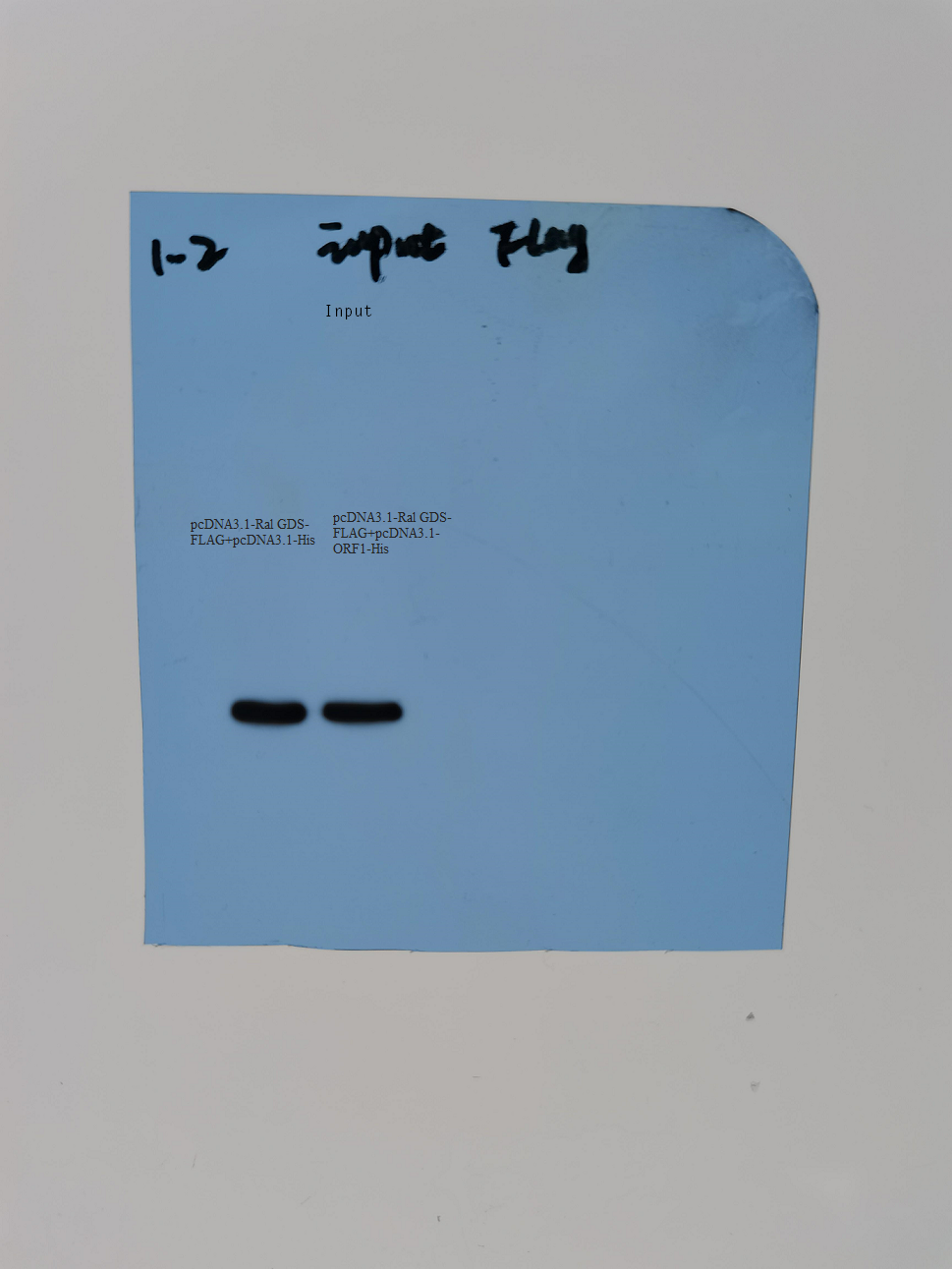

Supplement: Supplementary file 3 — Additional file 3: Figure S5 Co-immunoprecipitation of Cap interacted with RalGDS. HEK 293 Graham cells were co-transfected with pcDNA3.1-RalGDS-FLAG and pcDNA3.1-His (5a); pcDNA3.1-RalGDS-FLAG and pcDNA3.1-ORF1-His (5b); pcDNA3.1-FLAG and pcDNA3.1-ORF1-His (5c); and pcDNA3.1-RalGDS-FLAG and pcDNA3.1-ORF1-His (5d). [file 12917_2021_2926_MOESM3_ESM.zip › Figure 5a.tif]

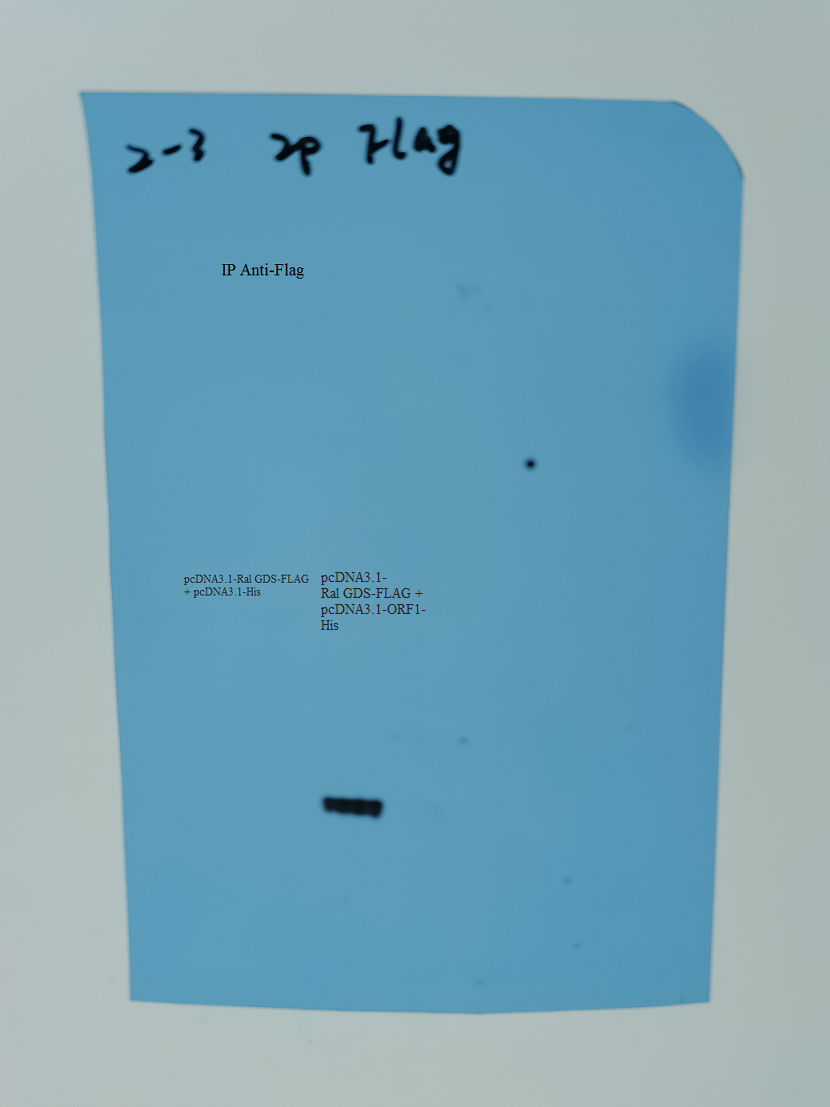

Supplement: Supplementary file 3 — Additional file 3: Figure S5 Co-immunoprecipitation of Cap interacted with RalGDS. HEK 293 Graham cells were co-transfected with pcDNA3.1-RalGDS-FLAG and pcDNA3.1-His (5a); pcDNA3.1-RalGDS-FLAG and pcDNA3.1-ORF1-His (5b); pcDNA3.1-FLAG and pcDNA3.1-ORF1-His (5c); and pcDNA3.1-RalGDS-FLAG and pcDNA3.1-ORF1-His (5d). [file 12917_2021_2926_MOESM3_ESM.zip › Figure 5b.tif]

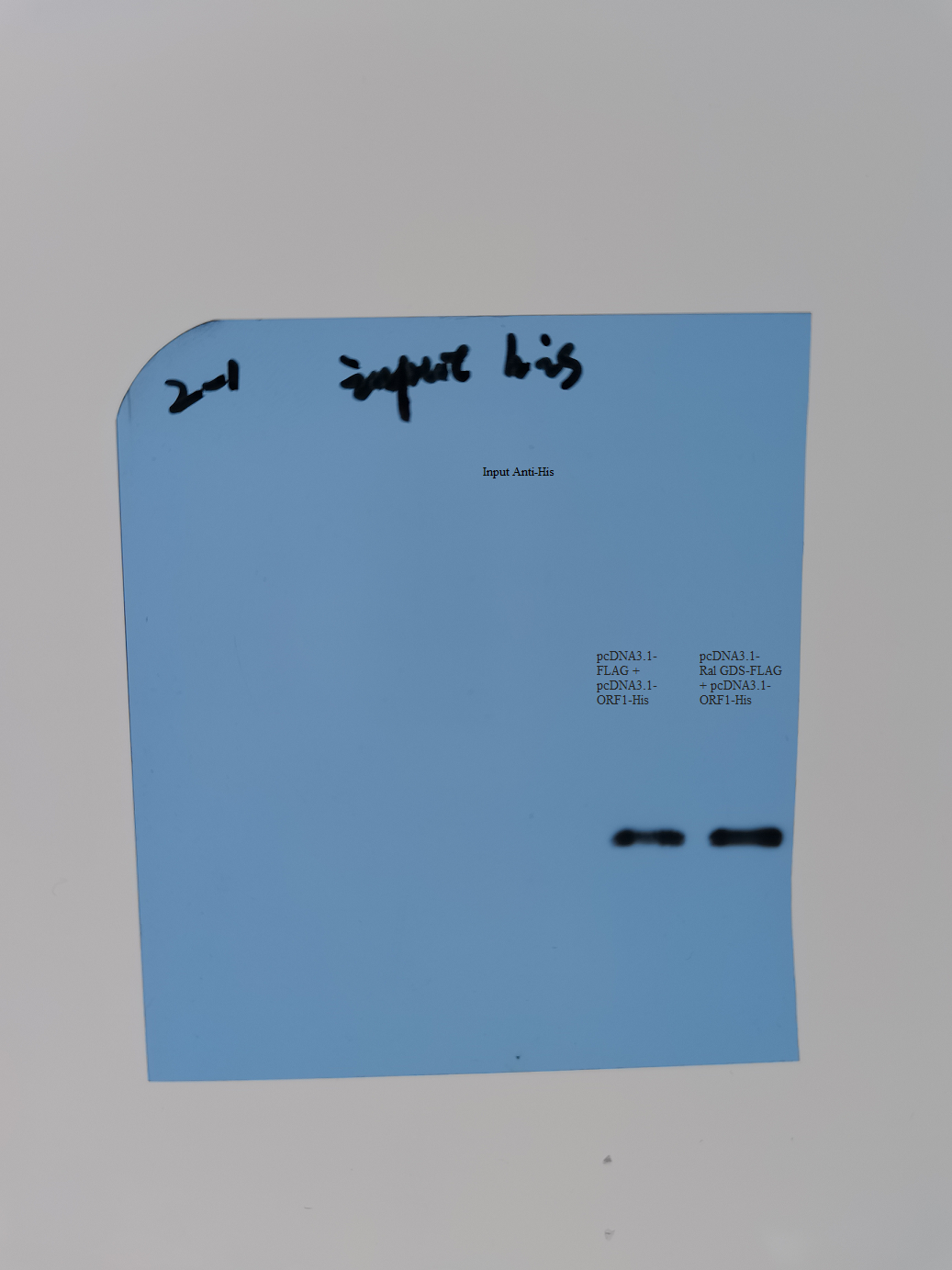

Supplement: Supplementary file 3 — Additional file 3: Figure S5 Co-immunoprecipitation of Cap interacted with RalGDS. HEK 293 Graham cells were co-transfected with pcDNA3.1-RalGDS-FLAG and pcDNA3.1-His (5a); pcDNA3.1-RalGDS-FLAG and pcDNA3.1-ORF1-His (5b); pcDNA3.1-FLAG and pcDNA3.1-ORF1-His (5c); and pcDNA3.1-RalGDS-FLAG and pcDNA3.1-ORF1-His (5d). [file 12917_2021_2926_MOESM3_ESM.zip › Figure 5c.tif]

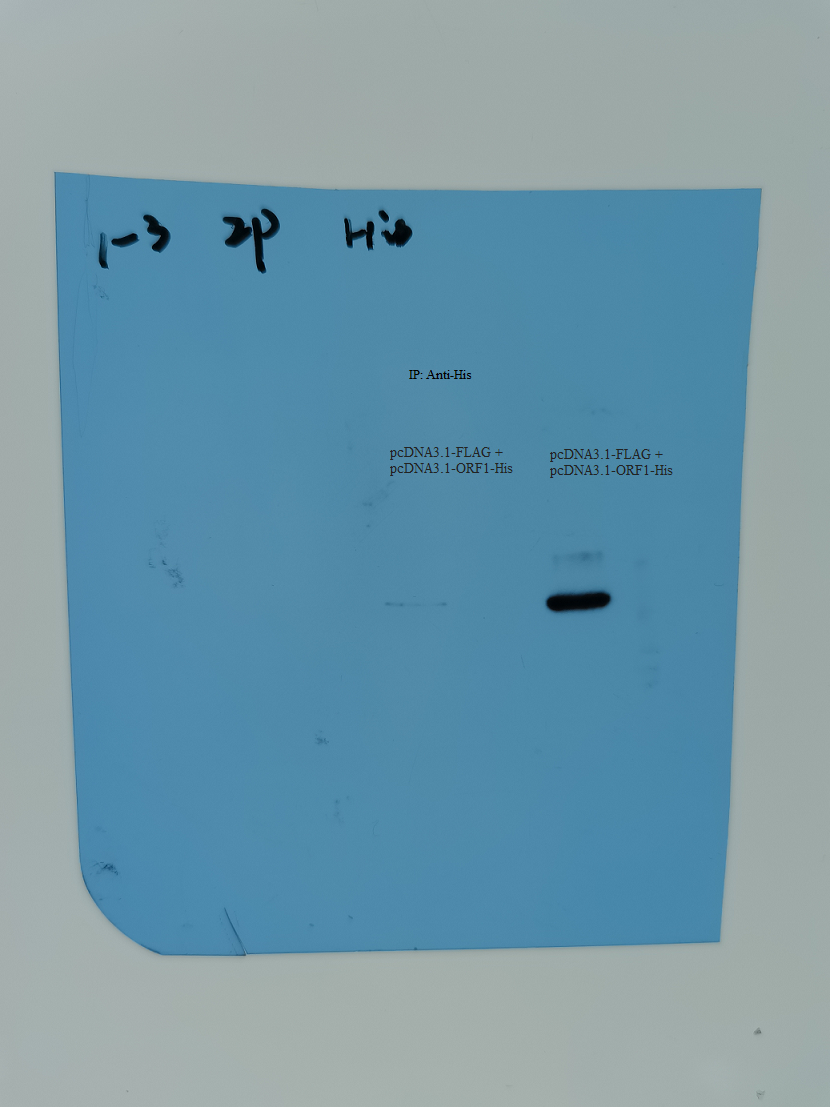

Supplement: Supplementary file 3 — Additional file 3: Figure S5 Co-immunoprecipitation of Cap interacted with RalGDS. HEK 293 Graham cells were co-transfected with pcDNA3.1-RalGDS-FLAG and pcDNA3.1-His (5a); pcDNA3.1-RalGDS-FLAG and pcDNA3.1-ORF1-His (5b); pcDNA3.1-FLAG and pcDNA3.1-ORF1-His (5c); and pcDNA3.1-RalGDS-FLAG and pcDNA3.1-ORF1-His (5d). [file 12917_2021_2926_MOESM3_ESM.zip › Figure 5d.tif]
